# Caught in the Middle: How and When Psychological Contract Breach by Subordinates Relates to Weekly Emotional Exhaustion of Supervisors

**DOI:** 10.3389/fpsyg.2020.464774

**Published:** 2021-01-13

**Authors:** Jeroen P. de Jong, Mike Clinton, Matthijs Bal, Beatrice Van Der Heijden

**Affiliations:** ^1^Institute for Management Research, Radboud University Nijmegen, Nijmegen, Netherlands; ^2^King’s College London, London, United Kingdom; ^3^Department of Management, University of Lincoln, Lincoln, United Kingdom; ^4^Faculty of Management, Open Universiteit, Heerlen, Netherlands; ^5^Department of Marketing, Innovation and Organisation, Ghent University, Ghent, Belgium; ^6^Business School, Hubei University, Wuhan, China; ^7^Kingston Business School, Kingston University, Kingston upon Thames, London, United Kingdom

**Keywords:** psychological contract, emotional exhaustion, performance pressure, supervisors, diary study

## Abstract

In psychological contract research, the side of the supervisor is strongly underexposed. However, supervisors are responsible for maintaining relationships with both their subordinates and senior management and are likely to be influenced by events unfolding in these relationships. In this study, we state that supervisor well-being may be affected by subordinates who fail to meet their obligations. This study adds to psychological contract research by developing an understanding of how and when subordinate psychological contract breach (PCB) is associated with supervisor emotional exhaustion. Through a weekly diary survey among 56 Dutch supervisors, we test hypotheses about the relationships between subordinate PCB and the emotional exhaustion of the supervisor, the mediating role of perceptions of performance pressure by the supervisor in this relationship, and the moderating role of i-deals between the supervisor and senior management. Multilevel analyses support the first two hypotheses, but contradictory to our expectations show that the positive association between subordinate PCB and the emotional exhaustion of the supervisor is strengthened when the supervisor has high levels of i-deals with senior management. We discuss the findings in relation to their contribution to psychological contract theory.

## Introduction

According to Mintzberg (1975, p. 49), the manager is the “heart of the organization,” having a major influence on follower attitudes, behavior, and performance (e.g., Podsakoff et al., 2006), as well as well-being (Skakon et al., 2010). They act as the linking pin between higher management and their followers, being the messenger of decisions of higher management on the one hand, and dependent on the efforts of followers in meeting organizational demands on the other hand. This implies that their role is accompanied by exchange relationships with subordinates as well as with senior managers (Coyle-Shapiro and Shore, 2007; Lee and Taylor, 2014). Middle managers are therefore particularly relevant in relation to the psychological contract—employees’ beliefs concerning mutual obligations between the employee and the organization (Rousseau, 1995)—due to their role as both employee of the organization and as organizational agent. However, despite the frequent critique of the employee-sided dominance of existence research (Guest, 1998; Hornung et al., 2009), and the recognition of the role of subordinates as causal agents in the development of supervisor well-being, behaviors, and outcomes (Shamir, 2007; Uhl-Bien et al., 2014), there is a serious lack of empirical work on supervisors’ experiences of psychological contract breach (PCB) by their subordinates.

In line with Dabos and Rousseau (2004), Chen et al. (2008), and Sherman and Morley (2020), we argue that supervisors develop mental models involving obligations from their followers and, just as with subordinates’ experiences of psychological contracts, these obligations can be fulfilled as well as breached. More specifically, supervisors can be affected by breach of obligations by their subordinates, for example, if they believe that the latter may have failed to meet a deadline, delivered work of decent quality, or supported the supervisor when important decisions were needed. In this study, building on psychology contract theory, we examine the impact of subordinate PCB, which we define as *the supervisor’s cognition that one’s subordinates have failed to meet one or more obligations within their psychological contract* (*cf.* Morrison and Robinson, 1997, p. 230), on supervisor emotional exhaustion, over the course of a number of weeks. Emotional exhaustion is commonly defined as “a consequence of intensive physical, affective, and cognitive strain” (Demerouti et al., 2001, p. 500), and is a critical predictor to work-related attitudes (Alarcon, 2011) and behavior such as job performance (Janssen et al., 2010). In addition to the impact emotional exhaustion can have for the supervisor, supervisor well-being has been found to trickle down and impact employees’ stress and well-being as well (Skakon et al., 2010; Huang et al., 2016).

**FIGURE 1:**
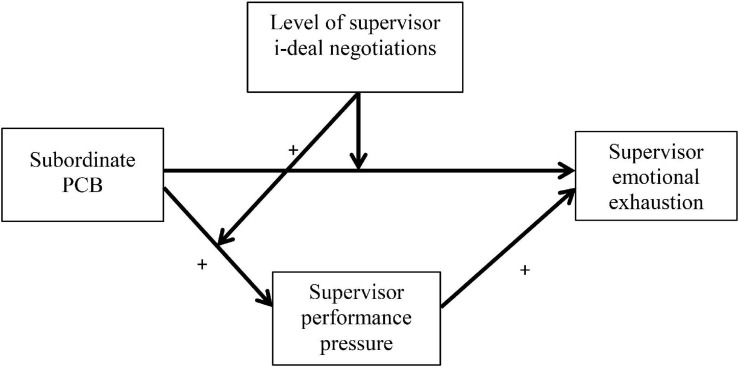
Theoretical model.

In addition, we will investigate how and when the relationship between subordinate PCB and supervisor well-being is more likely to manifest. We suggest that the relationship between subordinate PCB and supervisor emotional exhaustion is mediated by an increase in supervisor experiences of performance pressure. Because of their formal role in organizations, supervisors are responsible for “running the business” in their teams or departments and required to ensure that their teams or departments meet performance expectations, which can be closely monitored by their own managers (e.g., Ogbonna and Wilkinson, 2003). Therefore, we argue that these experiences of performance pressure, which are augmented by subordinate PCB, are likely to enhance their exhaustion (Giangreco and Peccei, 2005). Finally, we posit that the impact of job demands on emotional exhaustion is buffered through additionally negotiated resources from senior managers. In particular, one aspect of the job of supervisors is that they are in a mutually dependent relationship with their higher management (e.g., Mintzberg, 1975), and are in constant negotiation for additional resources that help them to fulfill their role expectations (Raes et al., 2011). More specifically, we argue that these negotiations take shape as i-deals; voluntary, personalized agreements of a non-standard nature that have been negotiated between the individual supervisor and higher management regarding terms that benefit both parties (Rousseau et al., 2006). We expect that i-deals buffer against the negative association between subordinate PCB and supervisor emotional exhaustion. Figure 1 shows the conceptual model of the current study.

This research contributes to the literature on psychological contract theory in several ways. First, our approach acknowledges the dual role of supervisors in psychological contracting; on the one hand, they communicate obligations to their subordinates, but, on the other hand, they are also recipients of promises of obligations from their subordinates (Lee and Taylor, 2014). To date, PCB by subordinates has been examined as (a) a reciprocated outcome of employee experiences of PCB by their organization (Coyle-Shapiro and Kessler, 2002; Dabos and Rousseau, 2004) or (b) as an antecedent of employee-level outcomes including organizational citizenship and performance (Tekleab and Taylor, 2003), lower levels of mentoring provision (Chen et al., 2008), and employee perceptions of PCB (Bordia et al., 2010). Therefore, we contribute to the scholarly literature in this field by substantiating that subordinate PCB also has implications for the supervisor him/herself.

Second, despite the large body of knowledge about employee PCB, including moderators and mediators that explain mechanisms between PCB and employee outcomes (for reviews, see Zhao et al., 2007; Bal et al., 2008), very little is known about the mechanisms underlying the impact of PCB at the supervisor level. By integrating role theory (Biddle, 1986) and psychological contract theory, we propose that the supervisory role is associated with specific expectations and responsibilities toward both followers and higher management that determine how and when subordinate PCB impacts supervisor well-being. Specifically, we further extend knowledge on PCB and its association with supervisor well-being by focusing on characteristics (performance pressure and supervisor i-deal negotiation) of their relationship with higher management as mechanisms that characterize their job and role characteristics.

## Theoretical Background

### Role Theory and the Supervisor

Role theory considers the way roles within organizations are defined and developed (Kahn et al., 1964). According to this theory, roles entail social expectations about the behavior of people occupying the roles that are or become attached to them (Biddle, 2013). Role theory proposes that persons are members of social positions such as supervisory or employee positions and based on their position, they hold expectations about their own behavior and the behavior of others (Biddle, 1986). This implies that people display different behaviors and have different expectations about the behaviors of themselves and others based on their role.

Role theory has been used to differentiate between the nature of supervisor and subordinate roles and their exchanges within organizations (Dienesch and Liden, 1986; Anicich and Hirsh, 2017). In recent decades, developments such as decentralization of responsibilities and the continuous pressure to change have elevated the importance of middle managers and supervisors in organizations (Balogun and Johnson, 2004; Heyden et al., 2018). Today, supervisors occupy a pivotal position and have several roles that together make their job, being responsible for implementing organizational strategies and policy, and at the same time shaping role expectations of their subordinates and controlling the performance within these roles (Harding et al., 2014). In contrast, the subordinate role is generally characterized by dependency on the supervisor in establishing role expectancies, and limited influence on role expectations of others including as the supervisor (Dienesch and Liden, 1986).

Supervisors also have roles that involve mutual dependencies with respect to both subordinates and higher management. This requires them to prioritize and balance the demands from both parties, making sense of both top-down and bottom-up communication processes (Lüscher and Lewis, 2008), and enter into negotiations over the resources they require from senior management (Bryant and Stensaker, 2011) and the resources they can make available to their subordinates (Rousseau, 2005). This degree of complexity, along with the need to be efficient and effective agents of organizational strategy and change, places substantial demands on supervisor roles (Madden, 2013). Within their subordinate role, supervisors are pressured to fulfill the role expectations of higher management. Moreover, as supervisors, they motivate their subordinates to fulfill their respective role expectations, which are critical to the fulfillment of expectations of the supervisor to higher management. Consequently, Bryant and Stensaker (2011) argue that middle managers are involved in frequent negotiation processes to manage all of their competing roles. They navigate these roles by seeking support and resources from higher management (Raes et al., 2011), and by negotiating with their subordinates about how they can best fulfill their role expectations (Dienesch and Liden, 1986).

### Subordinate Psychological Contract Breach

In terms of the psychological contract, the supervisory role includes a responsibility for managing employee contributions and overseeing the fulfillment of the employee side of the exchange relationship (Darden, 2019). However, this feature is not an obligation made toward their subordinates, yet rather an obligation made toward their organization, as part of the responsibilities that are inherent to their supervisory role. This implies that, similar to employees who have multiple, interdependent psychological contracts with different agents from the organization (Alcover et al., 2017), supervisors themselves have multiple, nested, and interdependent psychological contracts that result from ongoing negotiations with both higher management and subordinates. Their ability and motivation to fulfill an obligation in one psychological contract will depend upon the state of the obligations in the other relationship, and often upon the extent to which the other party has fulfilled their obligations to them as well. In previous research, the above-mentioned process has been examined via a “top-down” effect, whereby supervisor perceptions of organizational-level PCB lead to employee experiences of supervisory-level PCB, which, as a result, leads to poorer customer service (see Bordia et al., 2010). In the current study, we investigate a complimentary “bottom up” aspect of employee PCB, whereby the failure of employees to fulfill their obligations toward their supervisor has knock-on associations with variables at higher levels of the organization, in this particular case supervisor experiences of performance pressure and, consequently, supervisor emotional exhaustion in a particular week.

Similar to other team-level constructs, such as team OCB (e.g., Nielsen et al., 2012) and team proactivity (Lantz Friedrich et al., 2016), subordinate PCB reflects an assessment about the level of behavior expressed by the team as a whole. This means that not all team members need to show these behaviors, but the supervisor rates the general extent to which the team fulfills or breaches their obligations. These general assessments of group behavior are also in line with psychological contract research reasoning. Psychological contract theory generally considers the other party in the psychological contract as an “anthropomorphic” entity (e.g., “the organization,” Morrison and Robinson, 1997). Given the importance of teamwork for structuring work and achieving organizational outcomes (Mathieu et al., 2008), team leaders, while maintaining individual relations with subordinates, are primarily focused on team objectives and are evaluated for collective effectiveness (Carton and Cummings, 2012). Therefore, we propose that in evaluations of subordinate PCB, the subordinates of a supervisor can be considered as an entity as well.

## Hypotheses

### Subordinate PCB and Supervisor Emotional Exhaustion

According to psychological contract theory (Rousseau, 1995; Morrison and Robinson, 1997), a breached obligation entails an event where one party to the exchange relationship perceives that the other party has failed to fulfill an obligation that exists within that relationship, and in our specific case when a supervisor perceives that subordinate team members have breached one or more obligations to them. According to PC theory, this event is likely to elicit negative emotions, drain resources, and spur negative attitudes among the supervisor. Additionally, for both employees and supervisors, the psychological contract provides perceptions of predictability and control, trusting that the other party will follow through on their obligations (Gakovic and Tetrick, 2003). When the psychological contract is breached, this may decrease feelings of predictability and control, causing stress for the individual employee or supervisor (Gakovic and Tetrick, 2003) and potentially feelings of depression and anxiety (Conway and Briner, 2002).

Existing studies of the supervisor role would also support the view that subordinate PCB is a negative experience for supervisors that involves both negative emotional reactions and also remedial action that can further drain emotional and energetic resources (Darden, 2019). In a study among small business owners, Nadin and Williams (2011) found that experiencing severe subordinate PCB was followed by anger and upset, but also by concern for both damage to their authority as a leader and disruption to smooth running of business. In addition, Hallier and James (1997) suggested that when faced with subordinate PCB, middle managers’ behavior was motivated by attempts to protect the status, influence, and reputation associated with their managerial role as well.

So we build on existing psychological contract theory, that has identified negative emotions and a disruption to perceptions of control and predictability as outcomes of PCB, and from empirical work on supervisors’ experiences of subordinate PCB, which show that due to their role expectations, supervisors also engage in effortful remedial actions to maintain their role performance when subordinates fail to deliver on their obligations, we argue that subordinate PCB is related to supervisor emotional exhaustion during a particular week. Specifically we propose that subordinate PCB gives rise to supervisors’ concerns about the predictability and control over their associations with subordinates, and they may respond to these concerns by seeking to protect their own status and performance. While individuals are likely to use status and performance-protection strategies when faced with job demands, these strategies come with psychological costs, herewith draining the individual’s energy (Bakker and Demerouti, 2007). In sum, based on the outline given above, the following hypothesis is formulated:

H1: Subordinate PCB is positively related to supervisor emotional exhaustion.

### The Mediating Role of Performance Pressure

Continuing this logic regarding the negative relationship of subordinate PCB with emotional exhaustion, we introduce a key mediator to explain this relationship, that is, performance pressure. Performance pressure refers to “a belief that current performance is inadequate for achieving a desired goal” (Eisenberger and Aselage, 2009, p. 96). Managerial jobs are characterized by high levels of job demands and responsibilities (Janssen, 2001) and can face performance expectations from several stakeholders, including owners, customers, and employees. As explained above, we argue that subordinate PCB is likely to be a threat to the performance of the supervisor role, and in particular, it can lower a supervisor’s perceptions of control over whether they can achieve the desired goals associated with their role. We argue that this temporarily increases the performance pressure associated with their role, which would be supported by studies showing that lower job control associates with higher work pressure (e.g., Carayon and Zijlstra, 1999). Moreover, these expectations could give rise to role overload, situations in which people feel that they face too many expectations in light of the time available, their abilities, and available resources (Rizzo et al., 1970). Research consistently finds that role overload drains energy and positively associates to emotional exhaustion (Örtqvist and Wincent, 2006).

Moreover, supervisors are also dependent upon their own recent and current performance that lays the foundation for their future performance (Hambrick et al., 2005). When faced with subordinate PCB, and the associated performance pressure, supervisors will be motivated to engage in activities that will maintain their performance during a particular week (Eisenberger and Aselage, 2009). This notion suggests that, in the context of subordinate PCB, the supervisor needs higher levels of energy to respond actively, being an act of self-control (Baumeister et al., 2000) in order to restore the situation, while at the same time feeling the need to perform well in other supervisory tasks. The latter, in turn, is likely to emotionally exhaust the supervisor in a particular week. Otherwise stated, the reliance on the supervisor to clog the holes left by the subordinates as a result of subordinate PCB will entail more performance pressure (Ng et al., 2008). Moreover, previous research has shown that performance pressure on supervisors is associated with higher arousal and effort in completing a task (Gardner, 2012), as well as with higher emotional exhaustion (Knudsen et al., 2009). Therefore, we expect that:

H2: (a) Subordinate PCB is positively related to supervisor performance pressure; (b) performance pressure is positively related to supervisor emotional exhaustion; and (c) performance pressure mediates the positive association between subordinate PCB and supervisor emotional exhaustion.

### The Moderating Role of Supervisor i-Deal Negotiation

Finally, we argue that supervisors are particularly vulnerable for negative effects of subordinate PCB, partly due to them having to deal both with their subordinates and also with their organization (Coyle-Shapiro and Shore, 2007; Lee and Taylor, 2014). Raes et al. (2011) propose that integrative bargaining between top management and middle managers takes place as a response to competing interests, and, therefore, a better allocation of additional resources creates value for both parties. Moreover, as Bryant and Stensaker (2011) argue, supervisors engage in frequent negotiations with higher management about how their work should be organized in order to achieve goals. We therefore contend that supervisors have frequent negotiations with top management about the resources available to them, in order to meet their role expectations.

Rousseau (2005) refers to special conditions of employment that have been negotiated between an individual worker and his/her employer as i-deals. I-deals are individually negotiated, they can differ for each employee, even if they occupy the same position, vary in scope, and they should benefit both employer and employee (Rousseau et al., 2006). For employees, such i-deals provide them employment conditions and resources that satisfy their personal needs and preferences. Specifically, for employees with supervisory roles, these resources may include additional financial or manpower resources, personal development opportunities, or greater role flexibility (Shanock and Eisenberger, 2006) and autonomy (Knudsen et al., 2009), but also trust and support (Raes et al., 2011), and directions about goal attainment (Bryant and Stensaker, 2011). Moreover, due to changing personal needs and organizational contributions, employees may repeatedly bargain about features of their i-deals (Rousseau et al., 2006). Opportunities for ongoing i-deal negotiations are particularly present in social exchanges such as in supervisor–employer relationships, as successful i-deal negotiations signal a high-quality relationship and a basis for easier negotiations (Rousseau et al., 2009).

We suggest that the level of supervisor i-deal negotiations will weaken the negative association between subordinate PCB and supervisor emotional exhaustion, both directly and indirectly via performance pressure. Given the dual dependencies toward both subordinates and higher management that supervisors have to deal with in achieving their work goals, the opportunity to negotiate personal resources from higher management can balance perceptions of lower predictability and control that associate with subordinate PCB, help the supervisors to cope with the psychological costs associated with subordinate PCB, and better facilitate any remedial action necessary. Moreover, i-deal negotiations can decrease role overload, as additional resources would give the supervisor the confidence that he/she can meet their role expectations and thereby limit any influence on performance pressure. Indeed, support from those higher in managerial rank is found to buffer the negative effects of job demands (e.g., Kirmeyer and Dougherty, 1988; Schreurs et al., 2012), as support from higher management helps individuals to cope with job demands. For example, if subordinates fail to support a certain decision of their supervisor, experiencing trust by higher management could provide a boost in confidence in the decision by the supervisor. Furthermore, as supervisors consider subordinate PCB as damaging to their authority (Hallier and James, 1997; Nadin and Williams, 2011), supervisors may particularly seek support from leaders during such moments in helping them to exercise their hierarchical position (Hogg and Terry, 2000). Based on the theoretical outline given above, we formulated the following moderation and moderated mediation hypothesis:

H3: Supervisor i-deals moderate (a) the direct relationship between subordinate PCB and supervisor emotional exhaustion and (b) their indirect relationship via performance pressure, such that the effect in each case becomes weaker (less positive) as the level of i-deal negotiations becomes greater.

## Materials and Methods

### Design

Instead of single-shot transactions, psychological contracts involve processes of subsequent exchanges of contributions and inducements (Conway and Briner, 2005). Subordinate PCB is likely to vary over the course of weeks, as a natural rhythm of working concerns the working week (e.g., Bakker and Bal, 2010; Bal et al., 2017). During the working week, supervisors manage projects, deadlines, and people, and may develop evaluations of how their team is performing and whether the team is fulfilling its obligations in lieu of the tasks and deadlines for a particular week. Hence, the week as a unit of measurement for psychological contract evaluations is consistent with previous research on psychological contract dynamics and adds to the collection of existing diary research on the psychological contract from the employee perspective (e.g., Conway and Briner, 2002; Solinger et al., 2016; Bal et al., 2017). In addition, employee/supervisor job strain is also a volatile concept that can vary on a weekly or even a daily basis (e.g., Totterdell et al., 2006). Moreover, supervisors also use day-to-day negotiations and interactions to shape their roles (Rouleau, 2005; Bryant and Stensaker, 2011). To acknowledge the dynamic nature of the processes described above and to model the effects identified at the within-person level of analysis, this study adopts a weekly-diary methodology involving a sample of supervisors. To capture weekly variance of psychological contracts, supervisor emotional exhaustion, and i-deals, we implemented a weekly diary design collecting data over four consecutive weeks.

For this study, we did not seek approval from an ethical committee. We did not conduct a medical study, and our participants were not subject to procedures or were required to follow rules of behavior. Therefore, the research that we performed was exempt from such approval in the country in which the study was performed (the Netherlands) and by the institution leading this project (the Open University of the Netherlands). All research participants were informed in an introductory explanation of the survey that their participation was completely voluntary and that they could quit at any time, and that they would formally agree to participate in the research by filling out the survey, thereby giving informed consent if they chose to participate.

### Procedure and Sample

In 2015, a total of 56 supervisors from five Dutch organizations were recruited to participate, including a Dutch ministry (*n* = 28), a temporary work agency (*n* = 7), an energy company (*n* = 3), an insurance company (*n* = 8), and a chain of restaurants (*n* = 10). Initially, 73 supervisors were approached by the representatives of the five Dutch organizations to participate. Due to time constraints, 17 supervisors dropped out of the initial sample, resulting in a sample of 56 supervisors (77%) participating in the study. All participants were emailed on Thursday afternoon with a link to an online survey and started filling out the survey in the same week they were approached. Thursday was chosen because it is fairly common in the Netherlands to have a day off on Friday, in case of leave, or to work from home, in case of time-spatial flexibility (Peters et al., 2009). A personal identification code was used to link the consecutive surveys.

Fifty-six supervisors took part in our study and 174 weeks of data were analyzed. On average, respondents filled in three surveys. 55% of the supervisors were men and had an average age of 45 years (SD = 9 years). Participants supervised a specific group of subordinates, with an average span of control of 19 subordinates (SD = 18), and were often responsible for managing a whole organizational unit, e.g., a restaurant or location of a temporary agency.

### Measures

This study used previously validated measures that were slightly adapted so that items reflected the subordinate or supervisor as appropriate and referred to one particular week. All variables were measured on a weekly basis over four consecutive weeks. To ensure that respondents referred to the past week in answering our questions, we added “this week…” to all items. The response categories ranged from: “totally disagree (1)” to “totally agree (5)” for all scales used in this study.

*Subordinate PCB* was measured using four slightly adapted items from the global measure of PCB developed by Robinson and Morrison (2000). In the introduction of the measure, we asked the supervisor to evaluate the extent to which his/her subordinates fulfilled promises to him/her during the past week. Items were: (a) “This week, I have not received everything promised to me by my subordinates in exchange for my contributions”; (b) “This week, my subordinates have broken many of their promises to me even though I’ve upheld my side of the deal”; (c) “This week, my subordinates have done an excellent job of fulfilling their promises to me” (reverse coded); and (d) “This week, I feel that my subordinates have come through in fulfilling the promises made to me” (reverse coded)^1^. Multilevel reliability at the within-person level, as computed by the formulas recommended by Geldhof et al. (2014), was acceptable: (α = 0.83; omega = 0.73; H = 0.80)^2^.

*Supervisor emotional exhaustion* was measured with four slightly adapted items from Wharton’s (1993) scale, for example: “This week I felt emotionally drained from my work.” The multilevel reliability of the scale was broadly acceptable (α = 0.71; omega = 0.67; H = 0.74).

*Supervisor performance pressure* was measured with three items based on the close monitoring scale of George and Zhou (2001). The items of their original scale focused on the direct supervisor, and we adapted the items to be more open in terms of the source of the pressure: “This week I felt great pressure to perform”; “This week I had no problems meeting the performance criteria set by higher management,” and “This week I had to give everything to meet the performance criteria.” The multilevel reliability of the scale was broadly acceptable (α = 0.72; omega = 0.59; H = 0.70).

*Supervisor i-deals* was measured with a slightly adapted version of the *ex post* i-deal scale developed by Rousseau et al. (2009) using two items: “This week I have been able to negotiate special arrangements that suit me personally,” and “This week I have been able to negotiate with my supervisor about specific arrangements about my work and responsibilities that suit me personally.” The multilevel reliability of the scale was acceptable (α = 0.86; omega = 0.85; H = 0.86).

*Control variables*: Supervisor PCB was controlled for at the within-person level to rule out any alternative interpretation of the findings that subordinate PCB is simply a direct social exchange of initial supervisor PCB and therefore a trivial extension of existing findings on PCB. Supervisor PCB was measured based on three items from Robinson and Morrison (2000) and an example item was: “This week I have done my best to fulfill promises to my employees (reverse coded).” The multilevel reliability of the scale was poor (α = 0.49; omega = 0.43; H = 0.43)^3^. Similar to how day of the week is often controlled for in daily diary studies, we controlled for the specific diary week using dummy variables at the within-person level to account for both good/bad week effects and practice effects over the study. Because our hypotheses were tested at the within-person level, we did not control for between-person variables. We ran additional models that controlled for gender and age, as from previous work it is known that gender and age can be associated with emotional exhaustion (Marchand et al., 2018), but found near identical results to those reported^4^.

### Data Analysis

The hypotheses were tested at the within-person level using multilevel structural equation modeling (MSEM) within Mplus 7 (Muthén and Muthén, 1998–2012). Following recommendations by Maas and Hox (2005), a sample size of 56 clusters at Level 2 is sufficient for multilevel analyses. MSEM in Mplus partitions the variance of the diary variables measured into between- and within-person latent components. To produce accurate within-person estimates, Mplus centers the latent within-person components of the variables to the group mean, and the between-person components of the variables are allowed to correlate. Accordingly, our results can be interpreted as follows: a positive relationship between a certain *x* and a *y* variable means that in weeks wherein a respondent reported levels of *x* that were higher than they did on average over the 4 weeks of the study, they reported higher levels of *y* as well.

Prior to the hypothesis testing, intra-class correlations were checked to ensure that an appropriate amount of variance in each of the study variables existed at both levels, which indeed appeared to be the case: subordinate PCB (between = 42%; within = 58%); supervisor emotional exhaustion (between = 33%; within = 67%); performance pressure (between = 58%; within = 42%); and i-deals (between = 24%; within = 76%). Tests of mediation and moderation were performed using the procedures advocated by Preacher et al. (2006, 2010).

## Results

### Confirmatory Factor Analyses

A measurement model containing the observed indicators for the variables presented in Figure 1 was examined first. As the main interest of this study was at the within-person level, and because the sample size was relatively small, items and their factor structure were only examined at the within-person level. Weekly indicators were group-mean centered and specified as within-person variables. Parallel person-level composites were added to the model at the between-level and were allowed to correlate, thus saturating the between-person part of the model. A robust maximum likelihood estimator was used within the confirmatory factor analyses to better accommodate the ordinal nature of the data. The theorized four-factor model, in which the items for each variable were loaded onto separate latent constructs fitted the data adequately [χ^2^ = 68.453, df = 59, *p* = 0.169; CFI = 0.98; TLI = 0.96; RMSEA = 0.030; sRMR = 0.071 (within) and 0.000 (between)], and better in comparison with a single-factor model, in which all items were loaded onto a single latent variable [χ^2^ = 325.117, df = 65, *p* < 0.001; CFI = 0.57; TLI = 0.04; RMSEA = 0.152; sRMR = 0.129 (within) and 0.000 (between)]. For further comparison, a series of three-factor models were also run, wherein, first, the items for subordinate PCB and performance pressure were loaded onto the same latent variable [χ^2^ = 109.904, df = 62, *p* < 0.001; CFI = 0.92; TLI = 0.80; RMSEA = 0.067; sRMR = 0.099 (within) and 0.000 (between)]. Second, the items for subordinate PCB and i-deals were loaded onto the same latent variable [χ^2^ = 160.737, df = 62, *p* < 0.001; CFI = 0.84; TLI = 0.59; RMSEA = 0.096; sRMR = 0.109 (within) and 0.000 (between)]. Third, the items for performance pressure and i-deals were loaded onto the same latent variable [χ^2^ = 166.698, df = 62, *p* < 0.001; CFI = 0.83; TLI = 0.56; RMSEA = 0.099; sRMR = 0.105 (within) and 0.000 (between)]. Fourth, the items for supervisor emotional exhaustion and i-deals were loaded onto the same latent variable [χ^2^ = 161.490, df = 62, *p* < 0.001; CFI = 0.83; TLI = 0.58; RMSEA = 0.096; sRMR = 0.106 (within) and 0.000 (between)]. Fifth, the items for performance pressure and supervisor emotional exhaustion were loaded onto the same latent variable [χ^2^ = 79.817, df = 62, *p* = 0.063; CFI = 0.97; TLI = 0.93; RMSEA = 0.041; sRMR = 0.071 (within) and 0.000 (between)]. Although this fifth model also seems to fit the data adequately, there is a significant chi-square difference (TRd = 14.675, df = 3, *p* = 0.002) between this and the theorized four-factor model. A sixth three-factor model in which items for subordinate PCB and supervisor emotional exhaustion were loaded onto the same latent variable did not terminate normally, which is indicative of poor fit.

Further, multiple two-factor models were tested. First, a model in which items for subordinate PCB and performance pressure were loaded onto one latent variable and items for i-deals and supervisor emotional exhaustion were loaded onto another latent variable was tested. [χ^2^ = 198.634, df = 64, *p* < 0.001; CFI = 0.78; TLI = 0.45; RMSEA = 0.110; sRMR = 0.126 (within) and 0.000 (between)]. Second, a model in which items for subordinate PCB and i-deals were loaded onto one latent variable and items for performance pressure and supervisor emotional exhaustion were loaded onto another latent variable was investigated [χ^2^ = 169.080, df = 64, *p* < 0.001; CFI = 0.83; TLI = 0.57; RMSEA = 0.097; sRMR = 0.109 (within) and 0.000 (between)]. Third, a model in which items for subordinate PCB and supervisor emotional exhaustion were loaded onto one latent variable and items for i-deals and performance pressure were loaded onto another latent variable was tested [χ^2^ = 227.351, df = 64, *p* < 0.001; CFI = 0.73; TLI = 0.34; RMSEA = 0.121; sRMR = 0.136 (within) and 0.000 (between)]. So none of the two- or three-factor CFA models were found to have equal or superior fit to the theorized four-factor CFA model.

### Hypotheses Tests

Composite variables were used to test the hypotheses with a maximum likelihood estimator. Means, standard deviations, and zero-order correlations are presented in Table 1 (including between-person associations that are not further discussed) and within-person estimates relating to the hypothesis tests are reported in Table 2.

**TABLE 1:** Descriptives and zero-order correlations.

		**Mean**	**SD_wp_**	**1**	**2**	**3**	**4**	**5**
1	Subordinate PCB	1.99	0.50		0.76**	0.10	−0.07	0.19
2	Supervisor PCB	1.83	0.45	0.34**		0.19	0.22	0.47
3	Performance pressure	2.59	0.65	0.30**	0.09		0.67**	−0.24
4	Emotional exhaustion	2.37	0.47	0.25**	0.18*	0.40**		−0.03
5	Supervisor i-deal negotiations	2.21	1.07	−0.02	0.03	−0.05	−0.10	
			SD_bp_	0.40	0.30	0.65	0.32	0.57

Notes: *p < 0.05. **p < 0.01. PCB, psychological contract breach; wp, within-person; bp, between-person. The between-person correlations are shown above the diagonal and the within-person correlations are shown below the diagonal. *n*_bp_ = 56. *n*_wp_ = 174.

**TABLE 2:** Estimates of within-person direct, indirect, and moderation effects.

	**Supervisor performance pressure**		**Supervisor emotional exhaustion**	
	**Std. Est. (β)**	***p***	**Std. Est. (β)**	***p***
*Non-mediation model*				
Subordinate PCB			0.210	0.021
*Mediation model*				
Subordinate PCB	0.379	0.006	0.129	0.340
Supervisor performance pressure			0.384	<0.001
*Indirect effects*				
Subordinate PCB → Supervisor performance pressure			0.146	0.021
			_bc_LLCI	_bc_ULCI
			0.040	0.219
*Moderation effects*				
Supervisor i-deal negotiations	0.093	0.706	−0.527	0.020
Subordinate PCB* Supervisor i-deal negotiations	−0.189	0.479	0.555	0.024

*Notes: PCB, psychological contract breach; Std. Est, standardized estimate; _bc_LLCI, unstandardized lower limit bias corrected 95% confidence interval; _bc_ULCI, unstandardized upper limit bias corrected 95% confidence interval. *n*^*between*^ = 56. *n*^*within*^ = 174. Control variables included in each model but not presented: Week, Supervisor PCB.*

First, a simple model examined the direct relationship between subordinate PCB and supervisor emotional exhaustion, accounting for the control variables. Both variables were regressed onto the control variables and the between-person level variables were allowed to correlate, so the model was fully saturated in terms of fit. A significant positive association was found between subordinate PCB and supervisor emotional exhaustion (β = 0.210, *p* < 0.05), herewith supporting Hypothesis 1.

Second, a moderated-mediation model was examined, with performance pressure added to the first model as a mediator and i-deals (and its interaction term with subordinate PCB) added as a moderator at the within-person level, as shown in Figure 1. The new variables were regressed onto the control variables at the within-person level and again the main study variables were allowed to correlate at the between-person level variables; these will not be discussed further. This model was found to fit the data well [χ^2^ = 8.548, df = 5; *p* = 0.129; CFI = 0.99; TLI = 0.94; RMSEA = 0.064; sRMR = 0.042 (within) and 0.006 (between)]. At the within-person level, significant positive associations were found between subordinate PCB and performance pressure (β = 0.379, *p* = 0.006) and between performance pressure and supervisor emotional exhaustion (β = 0.384, *p* < 0.001). An indirect effect between subordinate PCB and supervisor emotional exhaustion via performance pressure was found to be significant by both the *p*-value (β = 0.146, *p* = 0.021) and by bias-corrected 95% confidence intervals not containing a zero (unstandardized estimate = 0.112; LLCI = 0.040; ULCI = 0.219; produced via a Monte Carlo simulation used to accommodate the asymmetric nature of the sampling distribution of an indirect effect in multilevel models; Preacher and Selig, 2010). Hypotheses 2a, b, and c were therefore supported with our data.

**FIGURE 2:**
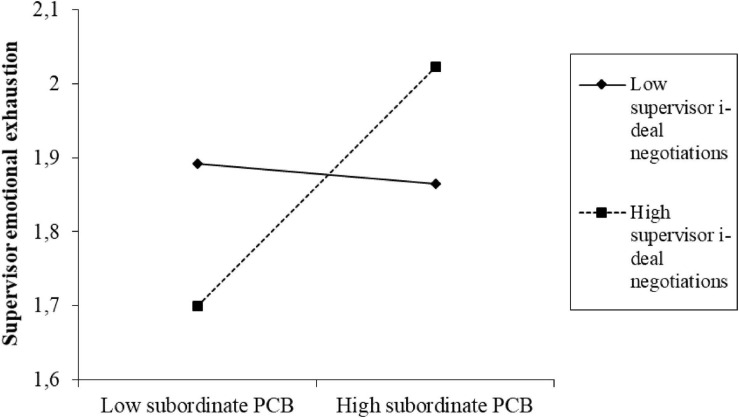
The effects of subordinate PCB on supervisor emotional exhaustion at high and low levels of supervisor i-deal negotiations.

The interaction term of subordinate PCB and i-deals was not significant in predicting performance pressure (β = −0.189, *p* = 0.479), and the indirect effect remained significant at both -1SD of the moderator (β = 0.382, *p* = 0.001) and at +1SD of the moderator (β = 0.287, *p* = 0.032), with no significant difference between these two effects found (β = 0.095, *p* = 0.486), which rejects H3b. The interaction of subordinate PCB and i-deals was significant in predicting emotional exhaustion (β = 0.555, *p* = 0.024). This interaction effect is represented visually in Figure 2 and suggests that, contrary to our expectations, the strength of the positive relationship between subordinate PCB and supervisor emotional exhaustion increases as the i-deals increase. A test of slopes indicates that the relationship is non-significant when the i-deals are low (−1 SD; unstandardized β = 0.002, *p* = 0.988) while it is significant and positive when the level of i-deals is high (+1 SD; unstandardized β = 0.242, *p* = 0.026). The difference between the slopes is significant (unstandardized βdiff = 0.240, *p* = 0.026), herewith rejecting Hypothesis 3a due to the opposing nature of the effect found.

## Discussion

The present study aimed to advance psychological contract theory by developing an understanding of how and when subordinate PCB associates with supervisor emotional exhaustion. Integrating propositions forwarded by psychological contract theory and role theory, we tested performance pressure perceived by the supervisor and negotiated i-deals between the supervisor and higher management as a mediator and moderator, respectively, to represent mechanisms that characterize the dual-dependencies of the supervisor role. The results of our study show that subordinate PCB positively associates with supervisor emotional exhaustion, and that performance pressure mediates this relationship. Furthermore, this study also finds that i-deals strengthened the positive association between subordinate PCB and supervisor emotional exhaustion, which runs counter to our hypothesis.

### Implications for Theory

The findings of this study offer contributions to our understanding of the psychological contracts of supervisors and of social exchange within organizations more generally. First, this empirical work provides evidence of a “bottom-up” (or upward) effect within the psychological contract. While always having been conceptualized as a process of two-way exchange and evidence of “top-down” (or downward) effects has been presented in relation to the psychological contract (Bordia et al., 2010), little work has been focused on examining the effects of breach and fulfillment by employees on the organizational agents that are a party to these exchange relationships as well (Darden, 2019). This study finds that within-person variation in subordinate PCB is positively related to supervisor emotional exhaustion. This finding is important, not in the least because of the well-documented effects of emotional exhaustion on wider health and functioning (Melamed et al., 2006; Halbesleben and Bowler, 2007) but also due to the limited work conducted on manager-level well-being in organizations (Pindek et al., 2020), which is likely to have distinctive antecedents (Quick et al., 2007; Knudsen et al., 2009). Evidence of this upward effect of the psychological contract on supervisor emotional exhaustion also offers support to perspectives that view employees as co-creators for leadership and leadership outcomes (Shamir, 2007).

For psychological contract theory, our focus on the supervisor brings new insights into the mechanisms that associate with well-being in a role other than the employee. Role theory proposes that roles entail social expectations about the behavior of those performing the role (Biddle, 2013). Because of their role, supervisors are expected to behave in ways that differ from employees, and have others behave toward them in ways that also differ. Accordingly, the effects of PCB on supervisors, and the context surrounding those effects, may similarly differ for supervisors compared with employees. In this light, the mediating role of performance pressure within this relationship is particularly interesting as it identifies a novel mechanism through which PCB influences outcomes, in addition to those identified at the employee level, such as violation and mistrust (Zhao et al., 2007). We argue that this mechanism, which is particularly apt for supervisors given the nature of their role, reflects a process in which subordinate PCB is associated with lower control over the achievement of desired goals and increased efforts to protect current performance of the supervisory role, however, at the cost of depleting energy and increasing his/her level of emotional exhaustion.

Second, the supervisory role includes frequent interactions with higher management about their performance goals and negotiations about resources that support goal achievement (Bryant and Stensaker, 2011; Raes et al., 2011). This study provides support for the notion of the interdependence of social exchanges within organizations and for the notion that the resources needed to fulfill obligations in one exchange relationship may be dependent on the resources gained from another exchange relationship (Foa and Foa, 1976; Shore et al., 2004). Specifically, this study finds that the level of i-deals between the supervisor and higher management strengthens the positive association between subordinate PCB and supervisor emotional exhaustion. This finding might seem counter-intuitive given the perspective that the receipt of more personal resources should better help an individual to cope with job demands, and also given findings from the i-deals literature which links personalized resources to reduced job stressors (Hornung et al., 2010). However, earlier empirical PCB research has shown that buffering effects of resources do not always occur (Bal et al., 2010), as the latter may also intensify the impact of PCB on certain outcomes. This is due to the nature of organizational resources being part of the social exchange framework between an employee and the organization (Rousseau, 1995; Tomprou et al., 2015). In other words, when individuals have access to resources at work, they are likely to feel obliged to reciprocate those favors (Rousseau et al., 2006). Thus, the provision of such negotiated resources may initially enable an individual (in our case a supervisor) to regain control over his/her role and to better cope with work demands (Rousseau, 1995), they may also create more vigilance and expectations on behalf of the organization with regard to the reciprocation by the individual (in our case a supervisor) for those additional resources. As suggested by the i-deals literature (Hornung et al., 2010), the provision of resources by an organization may indeed come “with strings attached,” which may put increased stress on supervisors to fulfill their obligations or engage in remedial action, and further raise the risk of reputational loss should they fail to do so. For supervisors, subordinate PCB exerts a heavy burden, as both exchanges with subordinates and higher management depletes energy, creating additional expectations and asking for additional actions to compensate the efforts from subordinates and engaging in negotiations with higher management.

For psychological contract theory, the interdependence observed in this study would advocate an extension of the nomological network of PCB, specifically, from one relating to a relatively narrow social exchange process between two parties to one in which the content and state of psychological contracts of a variety of stakeholders (e.g., senior managers, middle managers, employees, and colleagues) may be highly connected and dependent. Given our findings and that psychological contracts can develop between people occupying different roles in organizations (e.g., Rousseau, 1995; Lee and Taylor, 2014), psychological contract theory should further incorporate the role and position of people in organizations to better describe how people develop and respond to PCB.

### Practical Implications

It is well known that supervisors and middle managers have a complex job involving the management of relationships with their subordinates as well as with higher management (e.g., Hales, 2005). Supervisors are a critical factor in shaping the culture, operational processes, and subordinate performance in the organization. Our study shows that how managers perceive the social exchange within these relationships is linked to their well-being, emphasizing that the antecedents of supervisor well-being extend beyond the outcomes of their formal role performance to how they perceive fulfilling their role expectations. Considering the important role middle management has in organizations, higher management needs to be sensitive to the dual responsibilities of their middle management. This suggests that organizations should facilitate their leaders in such a way that they find means of reducing the performance pressure experienced by managers and provide managers with the tools to effectively manage the two-way psychological contract with subordinates. This may be done by means of training and development programs aimed at increasing supervisors’ competencies and their coping strategies, but most importantly, by providing support and developing trust in the capacity of their supervisors to deal with issues arising among their subordinates. Higher management should also be made more aware of the potential negative impact of negotiations with middle management and the potential for reciprocation pressure to create stress.

### Limitations

This study offers novel findings but there are several limitations to consider. First, while focused at the within person-level, the effects observed emanate from single-source data and from variables measured in the same weekly survey. Therefore, we cannot infer temporal order or completely rule out interpretations involving common-method variance for the inter-relationships between those variables, even though CFAs suggest it is unlikely (Podsakoff et al., 2003).

Second, our measure of subordinate PCB is a general assessment of the fulfillment of obligations of all subordinates. However, it is likely that there is some diversity in the extent to which different subordinates breach obligations toward the supervisor (Lee and Taylor, 2014). Research on LMX differentiation, for example, shows that supervisors form different quality exchange relationships with subordinates (Henderson et al., 2009) and that employees perceiving lower quality LMX-relations showed stronger negative reactions to PCB (Dulac et al., 2008). Similarly, not all breached obligations will have the same impact on supervisor emotional exhaustion. However, the measures and research design used in this study did not allow us to examine this diversity in subordinate PCB within groups or in the effects of different obligations.

Third, we did not directly test for competing mechanisms of association known to account for the impact of PCB beyond performance pressure. For instance, affective events theory (Weiss and Cropanzano, 1996) may offer account for an alternative explanation for the affective reactions of supervisors to subordinate PCB. Accordingly our model, while exploratory, remains under-specified in terms of mediating variables.

### Avenues for Future Research

On the basis of our study, we would suggest a number of avenues for future research. For example, future research could use supervisor–subordinate dyads (e.g., Breevaart et al., 2016) to include employee-rated leadership behaviors as outcome variables. A particularly interesting avenue would focus on the extent to which subordinate PCB associates to supervisor PCB as perceived by subordinates, using sample sizes that yield the required power to conduct reliable analyses (Ohly et al., 2010). This could be developed into direct assessment of the social exchange underlying psychological contract theory, as well as the conditions under which this exchange develops.

The model tested could be expanded in a number of ways. One way is to consider additional outcomes of subordinate PCB breach. For example, it would be interesting to see if supervisors become more transactional in their leadership behaviors following subordinate PCB, more vigilant of subordinate PCB, and more punitive of it, over time. Another way to expand the model would be to consider more details about i-deal negotiations and further boundary conditions, as researchers have found that not all i-deals carry along additional performance expectations (Hornung et al., 2010). For example, further research is needed to distinguish between the specific content of different types of i-deals in order to better investigate their predictive validity. Future work should incorporate separate variable measurement across time and across appropriate types of respondents.

## Conclusion

Through examining subordinate PCB and its relationship with supervisor-related outcomes at a weekly level, we showed that the effects of breaching obligations do not only trickle down from the organization to the employee, but can also be conceptualized as a bottom-up construct. We find that breached obligations by subordinates associate with negative supervisor outcomes, and particularly emotional exhaustion. In addition, we argue, and demonstrate through the mediating role of performance pressure and moderating role of i-deals, that the “how’s” and “when’s” of PCB can be influenced by role-specific attributes of the two parties to the deal, and specifically that explanations of supervisor reactions require an understanding of that role within the organization, which has also not received sufficient attention in the psychological contract literature.

## Data Availability Statement

The datasets generated for this study are available on request to the corresponding author.

## Ethics Statement

At the time this study was conducted (2015), there was no ethics committee in place at the university where the first author (JJ) was employed (the Open University of the Netherlands). We did, however, use a consent procedure for this study:

– Respondents were informed about the goal of the study.
– They were informed about the fact that the data collected in the study were used for scientific research.
– They were made aware of the fact that they could opt out of the study at any time.

Respondents consented to the study by clicking a box stating that they agreed with all statements above.

## Author Contributions

JJ contributed to theorizing, analyzing, writing, and coordinating. MC contributed to theorizing, analyzing, and writing. MB and BV contributed to theorizing and writing. All authors contributed to the article and approved the submitted version.

## Conflict of Interest

The authors declare that the research was conducted in the absence of any commercial or financial relationships that could be construed as a potential conflict of interest.

## Footnotes

1 The item “Almost all the promises made by my employer during recruitment have been kept so far (reversed)” from the original scale by Robinson and Morrison (2000) was dropped because a small pilot study revealed that it could be interpreted as a breach of legal contract rather than PCB.

2 Omega refers to the composite reliability and “represents the ratio of a scale’s estimated true score variance relative to its total variance” (Geldhof et al., 2014, p. 73) and H refers to maximal reliability and comprises “an alternative to comparing true score variance to the variance of a unit-weighted scale” (ibid, p. 73).

3 Due to the low reliability of the supervisor PCB measure, all analyses were run again but without controlling for this measure. No substantive differences in findings were noted. Therefore, we retained the measure for the main results presented, as it, at least partially, removes the social exchange interpretation detailed in the main text.

4 These results as well as a correlation table including these control variables can be obtained from the corresponding author.
